# Does body mass index impact assisted reproductive technology treatment outcomes in gestational carriers

**DOI:** 10.1186/s12958-020-00602-2

**Published:** 2020-05-02

**Authors:** Noga Fuchs Weizman, Miranda K. Defer, Janice Montbriand, Julia M. Pasquale, Adina Silver, Clifford L. Librach

**Affiliations:** 1 CReATe Fertility Centre, 790 Bay St #1100, Toronto, ON M5G 1N8 Canada; 2 Department of Anesthesia, Sunnybrook Health Sciences Centre, 2075 Bayview Avenue, Room M3200, Toronto, ON M4N 3M5 Canada; 3 Department of Obstetrics and Gynecology; Faculty of Medicine, University of Toronto, 123 Edward St. Suite 1200, Toronto, ON M5G 1E2 Canada

**Keywords:** ART outcomes, Obesity, BMI, Gestational carriers, Clinical pregnancy rate, Infertility treatment outcomes, Miscarriage rate, Live birth rate

## Abstract

**Background:**

The purpose of this study was to assess whether increased body mass index (BMI) negatively affects assisted reproductive technology (ART) outcomes among gestational carriers.

**Methods:**

A retrospective matched case-control cohort, including all gestational carrier (GC) cycles performed at CReATe Fertility Centre (Toronto, ON, Canada) between 2003 and 2016.

**Setting:**

A Canadian fertility clinic, with a large surrogacy program.

**Patients:**

All gestational carriers that had undergone a cycle completed to a transfer at our clinic, and had BMI and outcome data available, were matched by BMI to infertile patients treated at our clinic during the same years provided they had undergone a cycle completed to a transfer, and had outcomes data available.

**Interventions:**

None.

**Main outcome measures:**

Clinical pregnancies rates, miscarriage rates and live birth rates.

**Results:**

BMI was not a reliable prediction factor of any of the measured outcomes. Importantly, the gestational carrier population had better outcomes and a significantly lower overall incidence of maternal, fetal and neonatal complications when compared with infertile patients, treated at our clinic during the same years.

**Conclusion:**

BMI is not a reliable predictor of outcomes among gestational carriers.

## Background

Gestational carriers (GCs) are generally healthy women with proven fertility and a good obstetric history, who choose to carry a baby not genetically related to them for intended parent(s) [1]. Third-party reproduction is on the rise; in Canada, between 2001 and 2012, the number of births to GCs has increased six fold, and the proportion of ART births that involved a GC during the same years, doubled from 0.9 to 1.7% [2]. In the US, 2.4% of all IVF cycles performed between 2010 and 2014 involved GCs, resulting in 10,009 infants born [3].

Body Mass Index (BMI) measures body fat composite, based on height and weight. The World Health Organization (WHO) defines obesity as a BMI of 30 or greater. The abnormal fat accumulation in obese individuals can negatively impact their health in many ways, including an increased risk for vascular disease, diabetes and malignancies [4, 5]. As a result, obesity is an increasing public health concern and has been termed a ‘global pandemic’ [6].

Elevated maternal pre-pregnancy BMI can lead to a higher risk of maternal and fetal morbidities and mortality [7–13]. It is controversial whether or not obesity impacts the response to assisted reproductive technology (ART) treatments [14–18]. Some authors cite lower implantation rates (IR), clinical pregnancy rates (CPR) and live birth rates (LBR), as well as higher miscarriage rates among the obese population [17, 19–25], whereas others do not report any differences [26–29]. To better understand the impact obesity has on ART outcome and success rates, the effect on egg and embryo quality should be assessed separately from the effect on the uterine environment.

Measuring outcomes in GCs can enhance our understanding of the effect increasing BMI has on the uterine environment, while controlling for other confounding factors resulting from underlying causes of infertility. To further enhance our findings, and make them generalizable to other clinics, we chose to compare these outcomes to those achieved by our general infertile population at the clinic, during the same time period. Since, by design, surrogates have proven fertility and a favorable obstetric history, we hypothesized that per BMI they would achieve better ART outcomes, than infertile patients.

Importantly, studying the effect increased BMI has on outcomes among GCs, would assist in better defining screening policies for GCs; while some advocate using BMI as an exclusion criterion for GCs, this is not supported by the official American Society for Reproductive Medicine (ASRM) guidelines [30]. Previous studies, evaluating the impact obesity has on pregnancy outcomes in GCs have yielded conflicting results [31, 32].

BMI, per se, may not be the major determining factor influencing the outcome of infertility treatments; other metabolic and endocrinology factors may be at play [33–35]. This study was designed to assess whether BMI is a predictor of pregnancy outcomes among GCs, and how these outcomes differ from outcomes among the general infertile population exposed to similar conditions.

## Methods

This retrospective analysis received approval from the University of Toronto Research Ethics Board (#33894).

### Patient population

All patients that had utilized GCs (cases) between 2003 and 2016, at a private fertility clinic, were included in the study. All cases were matched with controls, which were patients that had undergone infertility treatments at the same fertility center during the same years.

### Inclusion and exclusion criteria

All cycles with missing BMI information or missing outcome data were excluded from the study population. Patients with infertility diagnosis or characteristics that could affect implantation and/or pregnancy outcomes (such as repeat implantation failure (RIF), repeat pregnancy loss (RPL) or thin endometrium prior to embryo transfer) were excluded from the control cohort.

### Study design

A retrospective case-control study. Matching of a 1:1 ratio was based on BMI (of GCs in the case cohort and of intended parents in the control cohort) as well as on year of treatment, to control for changes that had occurred in our laboratory during the analyzed years.

An a priori power analysis was undertaken based on previous literature, where small to medium effect sizes were captured [36]. Therefore two sample size predictions were created, one for small effects and one for medium effects. Calculations were performed with G*power [37] assuming an alpha of 0.05, Beta of 0.80 and one degree of freedom. Outcomes showed that a range of 65–126 cycles in each group should allow sufficient power.

### Demographic information

Clinical charts were reviewed and data extracted included demographic parameters (such as age, gravity and parity), lifestyle factors (smoking, alcohol consumption, recreational drug use and exercise), past obstetric, medical and surgical history, infertility diagnosis, relevant ART treatment parameters (such as endometrial thickness upon progesterone commencement prior to embryo transfer, and fresh vs. frozen embryo transfers) and treatment outcomes (pregnancy, miscarriage and live birth rates). Pregnancy outcomes were collected from the Better Outcomes Registry & Network (BORN) database, which was created in 2009 to gather, interpret, disseminate and protect data on pregnancy, birth and childhood outcomes in the province of Ontario, Canada [38]. In cases prior to 2009, and also where outcomes were not available from BORN, such as non-Ontario birth cases, patients were contacted directly, and outcomes were recorded in their charts as part of their routine clinical care.

### Primary and secondary outcomes

Primary outcomes were clinical pregnancy rate, live birth rate, miscarriage rate as well as analyzing clinical pregnancy and live birth as binary (y/n) variables. Secondary outcomes included complications during or after pregnancy for the carrier of the pregnancy and for the fetus/newborn.

### Statistical analysis

Statistical analysis was performed using the Statistical Package for the Social Sciences (SPSS) software version 17. Normality of variables was examined through skew/kurtosis analysis, as well as visually. Outliers were captured using standardized scores and graphs, and sensitivity analyses were conducted with and without outliers. Outliers were removed from the final analysis when they were shown to affect the results of the applied models. Multi-co-linearity was assessed with variance inflation factor and tolerance. BMI was analyzed as a categorical variable, based on WHO definitions [4]. Outcomes were stratified by BMI. For continuous outcomes, average, standard deviations and relative risks were created within each group and sub strata. The differences were considered statistically significant if the 95% confidence intervals were not overlapping and did not cross one. Kruskal-Wallis tests were used to assess statistical significance. A Holm-Bonferroni correction was applied for multiple comparisons, to lower the chances of a false positive result. Categorical variables were represented by n and %. Odds ratios were calculated per BMI strata, and chi square or Fisher’s exact tests were used to assess statistical significance. Homogeneity of groups was compared by examining the medical backgrounds of the groups for continuous variables, and also by Levene’s test of homogeneity of variance as a rough guide. Linear regression was used to assess continuous outcomes (i.e. clinical pregnancy rate and live birth rate) while accounting for important potential confounders (age, number of previous pregnancies and previous pregnancy complications of the pregnancy carrier, and age of the woman contributing the oocytes to the cycle). Given the small sample size there was a limitation to the number of covariates that we could control for, so we chose to perform backwards selection, which allows the identification of the most parsimonious model while preserving power. All variables were entered in 1 block and backwards regression performed. The last remaining predictors were selected as covariates.

## Results

**Fig. 1:**
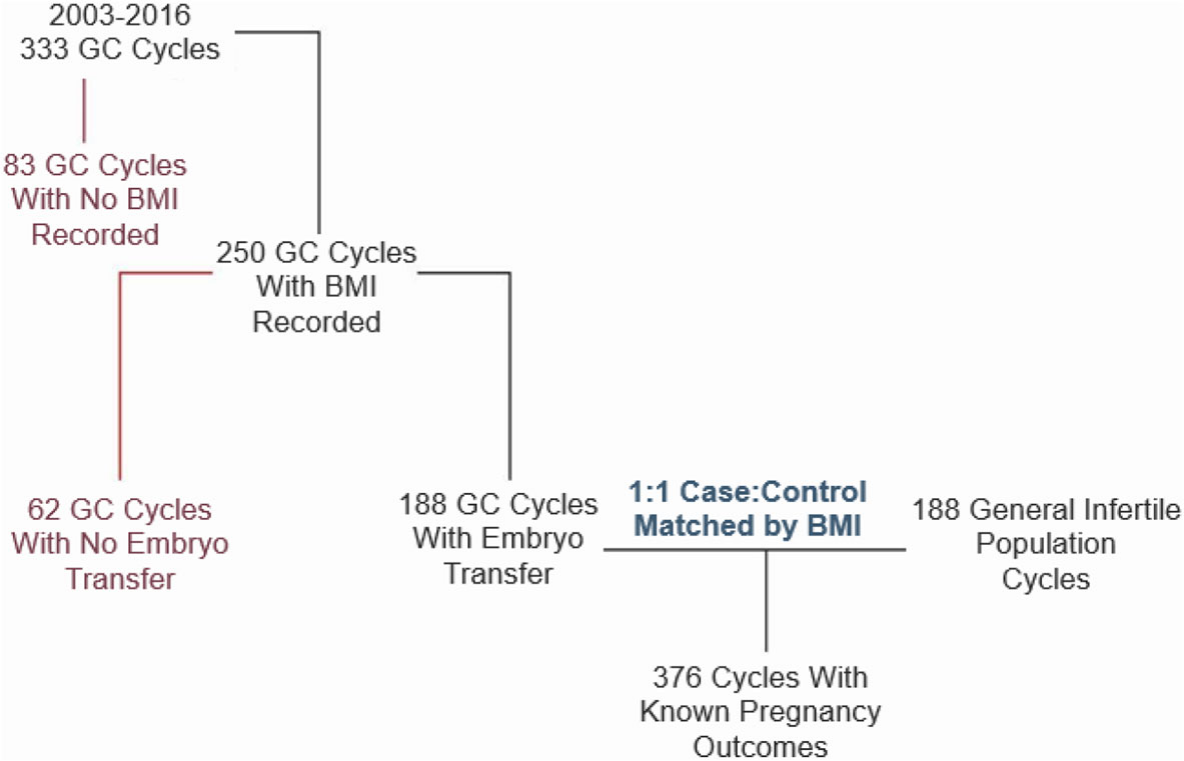
Participant inclusion and exclusion criteria flow diagram

There were 336 GC cycles performed at CReATe Fertility Centre between 2003 and 2016. Figure 1 depicts a flow chart of the review process leading to the final patient population of this study; following application of exclusion criteria, 188 GC cycles were included in the analysis and matched by BMI and year of treatment with the general infertile population treated during the same years, after applying exclusion criteria on this group as well. Eight GCs and 12 controls contributed more than 1 cycle to the final sample.

### Demographic characteristics, ART parameters and measurements of embryo quality

Table 1 depicts demographic characteristics of both cohorts, as well as relevant ART parameters. Women in our GC (case) cohort were significantly younger and had greater gravity (number of previous pregnancies) and parity (number of previous deliveries) than women in our general infertile (control) cohort, further more the oocytes utilized in our case cohort were retrieved from younger women. Cohorts did not differ in terms of endometrial thickness on day of progesterone commencement prior to embryo transfer. Fifty-three percent of the embryo transfers included in this study occurred on d3. Embryo grading did not differ significantly between the cohorts, nor did the number of embryos transferred in each transfer cycle (Table 2). Preimplantation Genetic Analysis was performed only on 2 embryos in the case cohort and on 6 in the control cohort. The average BMI within each category in cases and controls is depicted in Table S1.

**Table 1:** Demographic characteristics and ART parameters of cases and controls

Variables *Demographics*	N of records	Mean (Std. Dev)	*P* values
**Age of pregnancy carrier**			
GC cycles	187	31.83 (5.29)	< 0.05
Controls	185	37.38 (4.63)	
**Age of egg source**			
GC cycles	188	28.6 (5.5)	< 0.05
Controls	188	33.7 (10)	
**Number of previous pregnancies**			
GCs	188	3.28 (1.69)	< 0.05
Controls	188	0.94 (1.06)	
**Number of previous deliveries**			
GCs	188	2.32 (1.67)	< 0.05
Controls	188	0.18 (0.45)	
**Endometrial Thickness (mm)**			
GCs	179	10.13 (2.64)	NS
Controls	149	10.61 (1.95)

**Table 2:** Embryo characteristics and quality

Variables	N of records	Good Morphology	Fair Morphology	Poor Morphology	*X*^2^	*P* value
**Day 3 transfer**					0.427	0.8
GC cycles	91	42	43	6		
Controls	103	51	44	8		
**Day 5 transfer**					0.902	0.6
GC cycles	96	45	44	7		
Controls	76	38	35	3		
	N of records	N of embryos transferred per cycle (SD)	*P* value			
GC cycles	186	1.87 (0.6)	0.35			
Controls	178	1.8 (0.78)				

### Lifestyle factors

Table 3 depicts lifestyle characteristics, which were based on self-reporting.

**Table 3:** Lifestyle characteristics - proportions for cases and controls

Variables	GCs	Controls	*P* values
**Medication**	*n* = 112(%)	*n* = 177(%)	
Aspirin	0 (0)	4 (2.26)	NS
Anti-hypertensive therapy	0 (0)	2 (1.13)	NS
Psychiatric medication	18 (16.07)	11 (6.21)	< 0.05
None of the above medications	94 (83.93)	160 (90.40)	NS
**Smoking**	*n* = 91 (%)	*n* = 179 (%)	
Current	4 (4.4)	9 (5.03)	NS
Quit	8 (8.79)	23 (12.85)	NS
Never	79 (86.81)	147 (82.12)	NS
**Alcohol**^**a**^	*n* = 150 (%)	*n* = 179 (%)	
Light (3–6 drinks per week)	5 (3.33)	2 (1.12)	NS
Social (< 3 drinks per week)	99 (66.67)	80 (44.69)	< 0.05
Rarely	15 (10.00)	5 (2.79)	< 0.05
No	31 (20.67)	92 (51.4)	< 0.05
**Recreational Drug use**	*n* = 178 (%)	*n* = 177 (%)	
Current	2 (1.12)	10 (5.65)	< 0.05
Previous	2 (1.12)	0 (0)	NS
Never	174 (97.75)	167 (94.35)	NS
**Exercise**	*n* = 162 (%)	*n* = 156 (%)	
Yes	156 (96.30)	127 (81.41%)	< 0.05
No	5 (3.09)	29 (18.59)	< 0.05

^a^ [39]

### Measurements of group homogeneity

The GC cohort in this study was a more homogenous group, in terms of background, compared with controls; GCs were far less likely to present with medical comorbidities, prior to treatment, across all BMI groups (Table 4), this was further demonstrated by Levene’s test of Homogeneity of Variances (data not shown).

**Table 4:** Comorbidities of groups per stratified BMI

Variables	Normal weight	Overweight	Obese	Morbidly obese
**Thyroid disease**				
GCs	–	1 (2.2)	1 (2.3)	–
Controls	17 (30.3)	9 (14.8)	6 (30)	7 (38.9)
**Hypertension**				
GCs	–	–	–	–
Controls	1 (1.3)	3 (4.9)	1 (5)	–
**Depression/anxiety**				
GCs	–	–	–	–
Controls	5 (6.6)	3 (4.8)	1 (5)	–
**Diabetes**				
GCs	–	–	–	–
Controls	–	2 (3.3)	–	–
**Asthma**				
GCs	–	–	–	–
Controls	–	2 (3.3)	–	–

Data is presented as n (%)

### Prevalence of primary and secondary outcomes in cases and controls

Table 5 depicts comparisons between cases and controls for all primary and secondary outcomes in this study. Gestational carriers had higher implantation and clinical pregnancy rates as well as lower miscarriage rates, when compared with controls. Cohorts did not differ with regards to live birth rates. In addition, gestational carriers had fewer pregnancy and fetal complications, however mode of delivery did not differ significantly between cohorts. There were no differences in neonatal birth-weights between cohorts.

**Table 5:** Prevalence of primary and secondary study outcomes among cases and controls

**Primary Outcomes (%)**		**GCs**	**Controls**	***P Values***
Implantation Rate /embryos transferred		46.77	27.78	< 0.05
Clinical Pregnancy Rate/transfers		60.63	32.45	< 0.05
Miscarriage Rate/clinical pregnancy		15.79	31.15	< 0.05
Live Birth Rate/embryos transferred		49.91	53.74	NS
**Secondary Outcomes (%)**		**GCs**	**Controls**	***P Values***
Pregnancy Complications/clinical pregnancy		7.89	21.31	< 0.05
Fetal Complications/clinical pregnancy		3.50	16.39	< 0.05
Mode of Delivery/deliveries	*Vaginal*	62.50	28.58	NS
	*C-Section*	37.50	71.42	NS
Birth Weight/live birth	*LBW*	23.52	27.14	NS
	*LGA*	4.41	11.76	NS

*LBW* Low birth weight, *LGA* Large for gestational age

### Effect of BMI on primary outcomes

When examining whether BMI had an effect on clinical pregnancy rates or live birth rates, Kruskal-Wallis tests within each group were not significant for cases or controls (p > 0.05). The odds ratios for all 3 binary outcomes (clinical pregnancy, miscarriages and live births) were not affected by BMI (data not shown).

### Comparison of primary outcomes in cases and controls, per BMI strata

#### Clinical pregnancy

For all strata, there were higher percentages of clinical pregnancies in gestational carriers compared with controls. This reached significance in the normal weight (X^2^ = 7.23, *p* = 0.007), overweight (X^2^ = 16.7, *p* < 0.001) and obese groups (X^2^ = 14.2, *p* < 0.001). There was no difference found for the morbidly obese group (*p* = 0.5).

#### Live birth

There was a significant difference seen in the normal weight (X^2^ = 9.55, *p* = 0.002), overweight (X^2^ = 8.4, *p* = 0.004) and obese groups (X^2^ = 12.3, *p* < 0.001). There was no difference seen in the morbidly obese group (*p* = 0.44).

*All of the above remained significant after a Holm-Bonferroni correction was applied for multiple comparisons.

#### Miscarriage

Miscarriage rates did not differ between GC and Control in the normal weight (*p* = 0.19), overweight (*p* = 0.8) or morbidly obese (*p* = 0.9) groups. There was a trend towards higher miscarriages in obese controls (X^2^ = 8.9, *p* = 0.07). However, following a Holm-Bonferroni correction for multiple comparisons this was no longer significant (Table 6).

**Table 6:** Comparisons of primary outcomes between cohorts in each BMI strata

	Clinical pregnancy/embryo transfer	Live birth/embryo transfer	Miscarriage/clinical pregnancy
Normal weight			
GC	25/50 (50%)^a^	21/50 (42%)^a^	4/25 (16%)
Control	23/85 (27.1%)	15/85 (17.6%)	8/23 (34.8%)
Overweight			
GC	38/49 (77.6%)^a^	27/49 (55.1%)^a^	9/31^ (29.0%)
Control	25/64 (39.1%)	18/64 (28.1%)	6/23^ (26.1%)
Obese			
GC	37/56 (66.1%)^a^	34/56 (60.7%)^a^	3/32^ (9.4%)
Control	7/20 (35%)	3/20 (15.0%)	4/7 (57.1%)
Morbidly obese			
GC	14/33 (42.4%)	14/33 (42.4%)	2/13^ (15.4%)
Control	6/19 (31.6%)	6/19 (31.6%)	1/6 (16.7%)

Data presented as n (%); ^ adjusted for unknown live birth outcomes

^a^Chi Square or Fischer’s exact test shows significance (*p* < 0.05) - all remained significant after a Holm-Bonferroni correction was applied for multiple comparisons

After accounting for missing data, 8 % of the GC transfers were frozen embryo transfers and 11 % of the transfers among the matched controls were of frozen embryos. All cycles received similar luteal support as per routine in our clinic.

### Linear regression analysis

Neither clinical pregnancy rate, nor live birth rate were predicted by BMI of GCs when accounting for potential confounders, even when the confounders themselves differed between the groups (age, number of previous pregnancies, and previous pregnancy complications of the woman carrying the pregnancy, as well as age of the woman contributing the oocytes to the cycle) (*p* > 0.05) (Table 7).

**Table 7:** Linear regression model for continuous body mass index predicting outcome rates in GCs

	Standardized Beta Coefficient	*P*-values
*Clinical Pregnancy Rates*		
**BMI (continuous)**	**0.03**	**NS**
Age of woman carrying pregnancy	0.21	NS
Number Previous Pregnancies	−0.29	0.018
Previous Pregnancy Complications	0.06	NS
Age of woman contributing oocytes	−0.05	NS
*Live Birth Rates*		
**BMI (continuous)**	**0.08**	**NS**
Age of woman carrying pregnancy	−0.13	NS
Number Previous Pregnancies	−0.23	NS
Previous Pregnancy Complications	0.12	NS
Age of woman contributing oocytes	−0.36	0.04

*NS* Not Significant

## Discussion

Gestational surrogacy has been available since 1985 [1]. This has opened up options for women that are unable to carry a pregnancy (i.e. repeated implantation failure or repeated pregnancy loss), for women at risk for deterioration of their health if they conceive (i.e. malformations of the great vessels, kidney involvement of connective tissue diseases) and for patients wishing to conceive that do not have a uterus (agenesis of uterus, male patients). Cycles utilizing gestational carriers (GCs) are on the rise [2, 3]. GCs are usually younger than the general population seeking fertility solutions, and they have proven fertility, good obstetric history, and a relative scarcity of comorbidities, which was also the case in our study. A previous study concluded that previous proven fertility accounts for improved implantation rates in GC cycles, and is a positive prognostic factor of subsequent successful pregnancies [40]. Indeed, we were able to show improved implantation and clinical pregnancy rates as well as decreased miscarriage rates in our GC cohort compared with controls. It is important to note that the endometrial thickness prior to embryo transfer did not differ between cohorts, and that patients with RPL or RIF were excluded from the control cohort, thereby eliminating these factors as the driving forces for better outcomes among GCs. In this cohort, there was minimal utilization of PGT-A, and the oocytes utilized in GC cycles originated from younger women than those utilized in our control arm. However, when controlling for the age of the women contributing their oocytes to these cycles, BMI was still not a predictor for clinical pregnancy rates or live birth rates. Furthermore, it is well documented that the baseline aneuploidy rate in women younger than 35 is 30%, after which this rate increases significantly. Notably, in this study, the age of the oocyte source in both cohorts averaged below 35, thus cannot fully account for the remarkable differences in outcomes captured in this study [41]. It has been established that frozen embryo transfers yield superior results when compared with fresh embryo transfers [42]. Of note, in this cohort, the proportion of frozen embryo transfers was higher among the control population, which had worse outcomes per BMI strata. Furthermore, the cohorts also differed in prevalence of current recreational drug use, ‘social’ alcohol consumption and regular physical activity. These factors could not be controlled for in our analysis and thus represent a limitation of the retrospective design of this study.

Overweight and obesity are now dramatically on the rise, particularly in urban settings [43, 44]. In Canada, 25.8% of the population is obese [45]. Because of safety concerns and the perception that obesity might worsen the prognosis following ART treatments, some professional societies set BMI limits for eligibility for ART treatments [46–51]. The question whether BMI affects pregnancy outcomes in general, and ART outcomes specifically, is unresolved. Three previous studies that have assessed the effect BMI has on the uterine environment, by utilizing an oocyte donation model, yielded conflicting results [20, 26, 36]. However, such a model cannot control for other infertility factors that may contribute to or be impacted by increased BMI. Hence, using a GC model can help tease out such infertility factors, and increase the detection rate of the effects of increasing BMI on the uterine environment. A previous study looking at outcomes of 163 GC cycles was not able to capture differences in outcomes based on BMI category [32]. In a larger-scale study looking at 349 GC cycles, a threshold of BMI > 35 was found to be a significant predictor of worse outcomes [31].

Our study is the first to use the GC model while comparing with outcomes in the general infertile population. Comparing outcomes between GCs of different BMIs allowed us to control for other infertility factors associated with increased BMI. Our study is limited to the experience of one center. To make our findings more generalizable, we chose to compare outcomes among GCs to those achieved by our general infertile population of similar BMIs. An ideal study design would be a multi-center comparison of outcomes achieved by GCs from different BMI strata.

While most studies analyze pregnancies and live births as binary outcomes, it has been shown that when applicable, treating outcomes as continuous variables, specifically in smaller sample size studies, increases the power of the study [52]. The fact that we treated our primary outcomes as both continuous and binary variables adds strength to our analysis and provides a more in-depth interrogation of the effect BMI has on the tested outcomes. Further, since our sub-analysis concerns even smaller groups, we chose to apply a Holm-Bonferroni correction in pairwise comparisons to add robustness to the results. Indeed, we were able to show lower risks for maternal, fetal and neonatal complications among our GC population when compared with the general infertile population, matched by BMI. Furthermore, our GC population performed better for all measured outcomes, across most BMI strata (normal weight, overweight and obese), when compared with the general infertile population treated at our clinic. Since the GC population is inherently different than the general infertile population, in terms of age and favorable obstetric history, we performed linear regression to account for these potential confounders. In this regression model we were able to show that even after controlling for the above, BMI was not predictive of GC outcomes. In our cohort, BMI was not predictive of miscarriages; the miscarriage rate among our GC population was 11.6% and among our general infertile population it was 30.4%. A previous study found that increased BMI increases the risk for early miscarriages, as well as for repeat miscarriages [53]. These differences could be at least partially attributed to the fact that the women in the aforementioned cohort used their own eggs, and increased BMI could have potentially resulted in reduced oocyte quality, further demonstrating the importance of our study design.

Lastly, we had too few GCs with a BMI over 40 to make a judgment in that BMI range, future investigations should focus on the effect morbid obesity has on outcomes in GC cycles.

## Conclusions

In conclusion, in our study, BMI was not statistically or clinically predictive of ART outcomes or of pregnancy outcomes, among GCs. Due to the retrospective nature of our study and the limited sample size, these findings should be interpreted with caution and larger datasets are needed to further validate our findings. We suspect that other metabolic factors, which can be influenced by BMI, are at play, and may serve as better predictors for outcomes [31, 33, 35, 54, 55]. Currently the ASRM guidelines do not include increased BMI as an exclusion criterion for carrying a pregnancy as a surrogate [30]. Based on our study and previous literature, BMI should not be automatically applied as an exclusion criterion for GCs, when considering ART treatment. Consideration of previous obstetric history, and overall health are likely more predictive of adverse outcomes, and therefore, more important when screening a gestational carrier candidate. Future studies in this area should also look at other metabolic factors, such as lipid profiles and insulin resistance, which may be more helpful in identifying poor prognosis GC candidates, rather than BMI alone.

## Supplementary information

**Additional file 1: Table S1.** Average BMI within each category in cases and controls

## Data Availability

The datasets used and/or analyzed during the current study are available from the corresponding author on reasonable request.

## Abbreviations

ART: Assisted Reproductive Technology
ASRM: Assisted Society of Reproductive Medicine
BMI: Body mass index
BORN: Better Outcomes Registry & Network
CPR: Clinical pregnancy rates
GC: Gestational carrier
IR: Implantation rates
LBR: Live birth rates
WHO: World Health Organization
