# Supplementary material for: Does body mass index impact assisted reproductive technology treatment outcomes in gestational carriers
Source: Reprod Biol Endocrinol. 2020 May 2;18:35. doi: 10.1186/s12958-020-00602-2 (PMC7195786; doi:10.1186/s12958-020-00602-2)
Supplement: Supplementary file 1 — Additional file 1: Table S1. Average BMI within each category in cases and controls [file 12958_2020_602_MOESM1_ESM.docx]

**Table S1.** Average BMI within each category in cases and controls

|  | GCs | Controls |  |
| --- | --- | --- | --- |
| **BMI Category** | Mean (SD) | Mean (SD) | P values |
| **Normal Weight** | 21.85 (1.84) | 21.51 (1.98) | 0.32 |
| **Overweight** | 27.39 (1.37) | 27.19 (1.44) | 0.45 |
| **Obese** | 32.22 (1.47) | 32.16 (1.15) | 0.85 |
| **Morbidly Obese** | 37.4 (1.55) | 39.36 (4.21) | 0.07 |
